# Long-acting growth hormone therapy in paediatric growth hormone deficiency: reduced treatment burden and unproven motivation-related neuroendocrine effects

**DOI:** 10.3389/fendo.2026.1804285

**Published:** 2026-08-12

**Authors:** Rossella Gaudino, Franco Antoniazzi, Marco Zaffanello, Angelo Pietrobelli, Thomas Zoller, Ruggero Lanzafame, Giorgio Piacentini

**Affiliations:** 1 Department of Surgical Science, Dentistry, Gynecology and Paediatrics, Paediatric Unit, Verona University Medical School, Verona, Italy; 2 Pennington Biomedical Research Center, Baton Rouge, LA, United States

**Keywords:** adherence, dopamine, long-acting growth hormone, motivation, neuroendocrine regulation, paediatric growth hormone deficiency, reward system

## Abstract

Long-acting growth hormone (LAGH) formulations represent an important therapeutic advance in the management of growth hormone deficiency (GHD) because they reduce injection frequency and treatment burden and may improve adherence and patient-reported experience. Beyond these established advantages, we propose a hypothesis-generating framework in which motivation- and reward-related neuroendocrine pathways may interact with hypothalamic regulation of the growth hormone (GH) axis. This review summarises current knowledge on the neurobiological interplay among motivation, reward, stress, and GH secretion, with specific relevance to paediatric GHD and LAGH therapy. We discuss the roles of dopaminergic signalling, endogenous opioid systems, hypothalamic control of GH secretion, and the bidirectional interactions between GH and central neural circuits. Because direct clinical evidence in paediatric LAGH-treated populations remains limited, the proposed relationships should be interpreted as biologically plausible but not yet established causal mechanisms. Finally, we outline future translational and clinical studies needed to determine whether motivational state is associated with endocrine responses, treatment experience, or clinical outcomes beyond the well-documented benefit of improved adherence.

## Introduction

Growth hormone (GH) therapy remains a cornerstone of treatment for paediatric growth hormone deficiency (GHD), with well-established benefits for linear growth and clinically relevant metabolic outcomes (1).

In children, early and sustained exposure to adequate GH replacement is crucial for achieving optimal adult height and supporting normal metabolic programming. Nevertheless, conventional daily subcutaneous GH injections impose a significant long-term treatment burden on paediatric patients and their families. This burden is not limited to the physical discomfort of injections but also includes anticipatory anxiety, treatment fatigue, and emotional distress, all of which can negatively affect adherence and persistence over time. Daily GH injections may impose treatment burden, whereas weekly formulations have been associated with reduced treatment burden in paediatric GHD (2). Several once-weekly LAGH preparations have now received regulatory approval for paediatric GHD in major jurisdictions, including the United States and Europe. In Italy, AIFA documents and Nota 39 identify somatrogon as a growth hormone treatment for children and adolescents with growth failure due to insufficient GH secretion.

At present, the most evidence-supported clinical advantages of long-acting growth hormone (LAGH) in paediatric GHD include reduced injection frequency, treatment burden, and adherence, as well as improved treatment satisfaction and patient-reported experience (3, 4). Poor adherence to daily GH therapy has been recognised as a clinically relevant challenge in paediatric GHD, particularly because sustained exposure to GH is required during critical windows of growth. These challenges have driven the development of long-acting growth hormone (LAGH) formulations, which enable weekly or less-frequent administration while maintaining efficacy comparable to daily GH. Recent international consensus statements now recognise LAGH as a valid therapeutic option in paediatric GHD, emphasising potential advantages in adherence, treatment satisfaction, and quality of life. While improved adherence is the most immediate and evidence-based benefit of LAGH therapy, it may not fully capture the broader behavioural and neuroendocrine consequences of reducing injection burden (3).

Repeated daily injections may constitute a treatment-related stressor in some children receiving rhGH therapy, particularly in the presence of needle fear, injection-related discomfort, or chronic therapy-related distress (5). Because stress is a well-established modulator of hypothalamic–pituitary function and growth-related neuroendocrine pathways (6), reduced injection burden with LAGH may plausibly influence emotional experience and stress perception in ways that could indirectly interact with GH regulation (2, 3, 6).

Motivation- and reward-related circuits, particularly mesolimbic dopaminergic pathways, are central to behavioural state, reward processing, and stress-related behavioural adaptation (7, 8). However, these pathways should be distinguished from hypothalamic and pituitary dopaminergic mechanisms involved in neuroendocrine regulation of the GHRH–GH axis (9, 10). Therefore, any proposed interaction between motivational state and GH regulation should be considered indirect, rather than evidence of direct motivational control of GH secretion.

### Scope, novelty, and critical appraisal approach

This article is intended as a hypothesis-generating perspective rather than a systematic review or clinical guideline. Although LAGH therapy has been extensively discussed in relation to pharmacokinetics, efficacy, safety, treatment burden, adherence, and patient-reported outcomes, the potential interface between reduced injection burden, motivational state, stress perception, reward-related circuitry, and GH neuroendocrine regulation has received limited direct attention.

Although LAGH therapy has been extensively discussed in relation to pharmacokinetics, efficacy, safety, treatment burden, adherence, and patient-reported outcomes, the potential interface between reduced injection burden, motivational state, stress perception, reward-related circuitry, and GH neuroendocrine regulation has received limited direct attention.

The added value of this review is in bringing together these areas of evidence: clinical data on LAGH-related treatment burden and adherence and mechanistic literature on stress, motivation, reward-related circuitry, and GH neuroendocrine regulation. The unique contribution of this review is to move beyond the established observation that LAGH may reduce treatment burden and improve adherence by proposing a structured, hypothesis-generating framework that considers treatment experience, stress perception, motivational context, and GH neuroendocrine regulation together. This framework does not establish a causal neuroendocrine mechanism, but it advances current understanding by identifying biologically plausible pathways and measurable variables that future translational and clinical studies can test.

Different review formats serve different methodological purposes, and narrative reviews may be appropriate for conceptual synthesis, provided that the rationale, evidence base, and limits of inference are transparently presented (11, 12). Therefore, the literature was interpreted according to the level of evidence supporting each proposed mechanistic link. Human physiological studies were considered the most directly relevant to GH regulation in clinical contexts, whereas animal, *in vitro*, and mechanistic neurobiological studies were used primarily to support biological plausibility rather than clinical causality.

Accordingly, the proposed framework does not assume that motivational state independently determines growth response to LAGH therapy. Instead, we distinguish three levels of inference: first, evidence-supported clinical effects of LAGH, particularly reduced injection frequency, reduced treatment burden, and potential improvements in adherence and patient-reported experience (3, 4); second, experimentally supported neuroendocrine interactions involving hypothalamic–pituitary regulation of GH secretion, including dopaminergic modulation of the GH–IGF-I axis and GH receptor signalling in dopaminergic neurons (9, 13–17); and speculative translational hypotheses linking treatment experience, stress perception, motivation, reward-related circuitry, and somatotropic regulation in children with GHD (7, 8). Importantly, the level does not imply that mesolimbic motivational dopamine neurons directly regulate pituitary GH secretion or that motivational state independently determines therapeutic response to LAGH.

### Neuroendocrine regulation of GH: dopaminergic and hypothalamic control

GH secretion is regulated by a complex neuroendocrine network centred on the hypothalamic balance between growth hormone–releasing hormone (GHRH) and somatostatin (18). GH pulses are sensitive to central nervous system inputs, allowing GH secretion to dynamically adapt to sleep–wake cycles, nutritional status, stress exposure, and behavioural context (19, 20).

Among these neuromodulators, dopamine has been implicated in GH regulation through hypothalamic and pituitary neuroendocrine mechanisms (9, 10, 13–15, 21, 22). Dopamine should be considered within anatomically and functionally distinct systems. In the context of GH regulation, dopaminergic effects are primarily relevant to hypothalamic–pituitary neuroendocrine mechanisms rather than to mesolimbic motivational circuitry. Experimental evidence from tyrosine hydroxylase-expressing neurons, D2 receptor knockout mice, and GHRH/somatostatin cell-specific models supports a role for dopaminergic signalling in GH secretion, the GH–IGF-I axis, and postnatal somatic growth in preclinical models (10, 15, 21, 22). Tuberoinfundibular dopamine neurons are also classical regulators of pituitary lactotroph function and prolactin secretion (23, 24), and should not be conflated with mesolimbic dopamine pathways involved in reward and motivation (7). Early *in vitro* studies suggest that dopamine may exert context-dependent effects on GH secretion, including stimulation of basal release and modulation of GHRH-related responses. These findings support a modulatory, rather than purely stimulatory, role for dopamine.

Overall, the available evidence supports dopamine as a modulatory contributor to the GHRH–GH axis, primarily through hypothalamic–pituitary neuroendocrine mechanisms (9, 10, 15). However, broader claims linking dopamine directly to motivational or emotional control of GH secretion require more direct experimental support than is currently available. Conversely, emerging evidence suggests that GH may signal back to selected dopaminergic neuronal populations involved in neuroendocrine regulation.

### Bidirectional crosstalk between GH and dopaminergic neurons

GH receptors are expressed in specific dopaminergic neuronal populations, and GH receptor signalling in these neurons may contribute to stress-related neuroendocrine regulation, including stress-induced prolactin release in male mice (16). More broadly, central GH signalling has been implicated in metabolic, neuroendocrine, stress-related, and behavioural adaptations (17). These findings support bidirectional interactions between the GH axis and selected dopaminergic neuronal populations, but they do not demonstrate that GH directly modulates mesolimbic motivational circuitry or LAGH response in paediatric GHD.

This distinction is important because dopaminergic systems are anatomically and functionally heterogeneous, with mesolimbic pathways implicated in motivation, reward processing, and stress-related behavioural adaptation (7, 8). Evidence for GH receptor signalling in selected dopaminergic neurons should therefore not be extrapolated to direct GH regulation of mesolimbic motivation or therapeutic response to LAGH (16). Accordingly, the following section considers motivation- and reward-related circuitry as a contextual, rather than directly causal, component of the proposed framework.

### Motivation, reward, and GH secretion

Within this context, motivation- and reward-related circuitry may be relevant to GH regulation primarily through indirect pathways, including stress perception, behavioural state, sleep, and metabolic signals (6, 25, 26). These circuits include mesolimbic dopaminergic pathways, commonly involving projections from the ventral tegmental area to the nucleus accumbens, which are functionally distinct from hypothalamic dopaminergic neurons involved in pituitary hormone regulation (7, 8, 23).

Therefore, the relevance of motivation-related circuits to GH secretion should be considered indirect and contextual, potentially mediated through stress perception, treatment experience, sleep, or metabolic signals rather than through a direct mesolimbic dopaminergic control of pituitary GH release (7, 8, 27).

Motivational state may interact with perceived stress and behavioural context; however, direct evidence that such states modify hypothalamic GH regulation or LAGH response in paediatric GHD is currently lacking. Within this framework, motivational context may be relevant indirectly because GH secretion is closely associated with sleep, energy balance, and tissue growth (8, 27).

Ghrelin represents a key molecular mediator at the intersection of metabolism, reward, and GH secretion. As a potent GH secretagogue, ghrelin stimulates GH release via both hypothalamic and pituitary mechanisms. In parallel, ghrelin activates dopaminergic neurons in the ventral tegmental area and enhances reward-related behaviours, including food-seeking and motivational drive (26). Ghrelin provides a biologically plausible link between metabolic state, GH secretagogue activity and reward-related dopaminergic circuitry. Nevertheless, evidence from ghrelin-related reward models does not establish that motivational state independently amplifies GH secretion or modifies endocrine response to LAGH in paediatric GHD (26, 28).

Endogenous opioid pathways have also been implicated in the neuroendocrine modulation of GH secretion, although the precise mechanisms and their relevance to paediatric LAGH therapy remain uncertain (9).

Because GH secretion is closely linked to sleep, energy balance, nutritional status, and stress-related hypothalamic regulation, motivational and reward-related states may be relevant mainly as contextual variables rather than direct determinants of GH secretion. In this framework, motivation may interact indirectly with GH regulation through treatment experience, stress perception, sleep quality, or metabolic signals (6, 25, 26).

**Figure 1:**
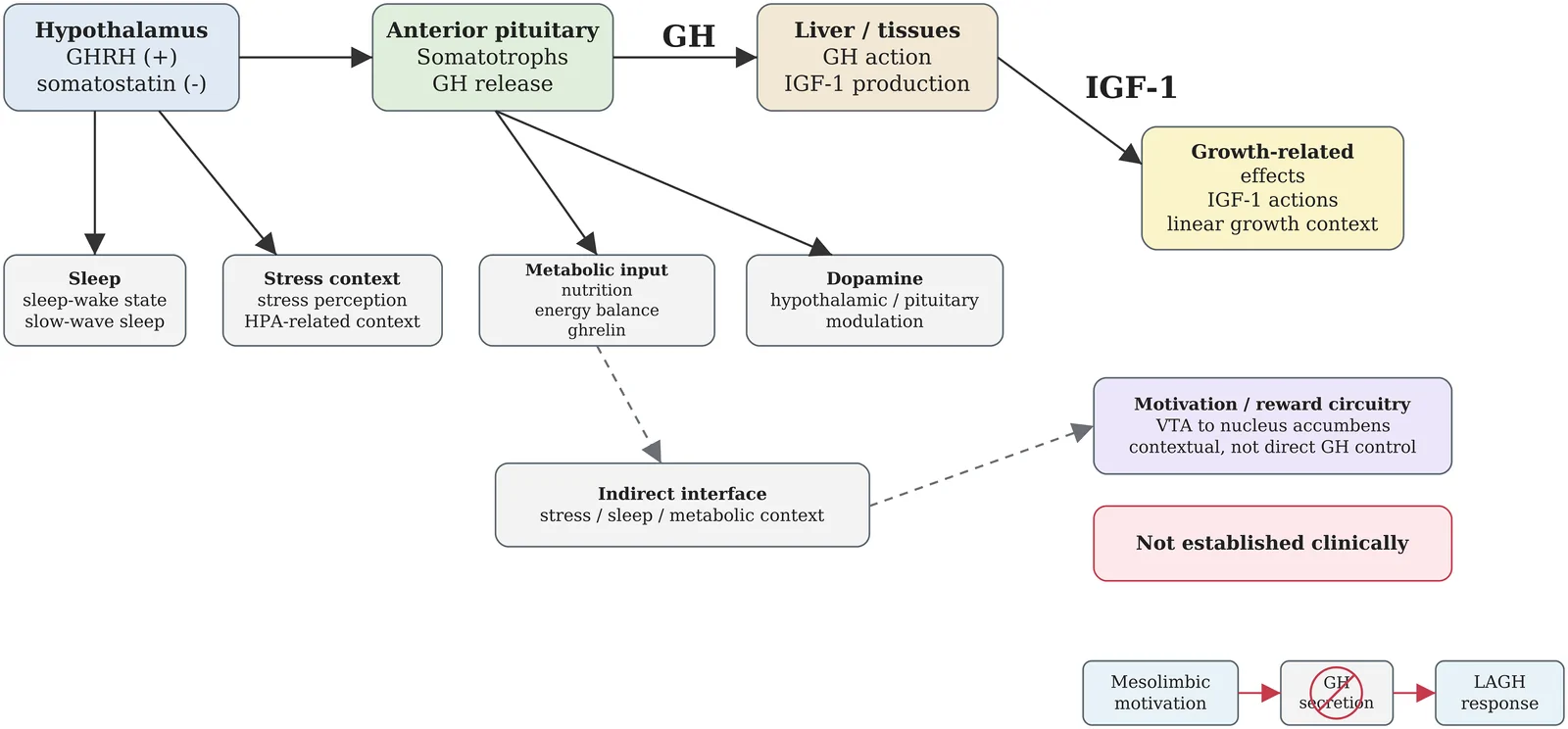
Established and contextual neuroendocrine pathways relevant to GH regulation. show established physiological links, including hypothalamic GHRH/somatostatin regulation of pituitary GH and downstream IGF-1 in liver and tissues. Sleep, stress, metabolic input, ghrelin, and hypothalamic–pituitary dopaminergic mechanisms may modulate this axis. indicate indirect or unproven links. Mesolimbic motivation and reward circuitry is separate, as it is functionally distinct from hypothalamic–pituitary dopaminergic pathways. Clinical evidence that mesolimbic motivation independently influences GH secretion or LAGH response in paediatric GHD is lacking (6–10, 13–18, 25, 26, 28).

Taken together, these pathways support biological plausibility, but they do not establish that motivational state independently determines endocrine response, growth outcomes, or therapeutic benefit during LAGH therapy. The established and contextual neuroendocrine pathways discussed in this section are summarised in **Figure 1**.

## Discussion

LAGH formulations reduce injection frequency and treatment burden, which may decrease injection-related anxiety and stress and improve treatment acceptance. In paediatric patients, these factors significantly influence treatment acceptance and long-term persistence (3). At present, this remains the best-supported mechanism by which LAGH may improve outcomes.

**Figure 2:**
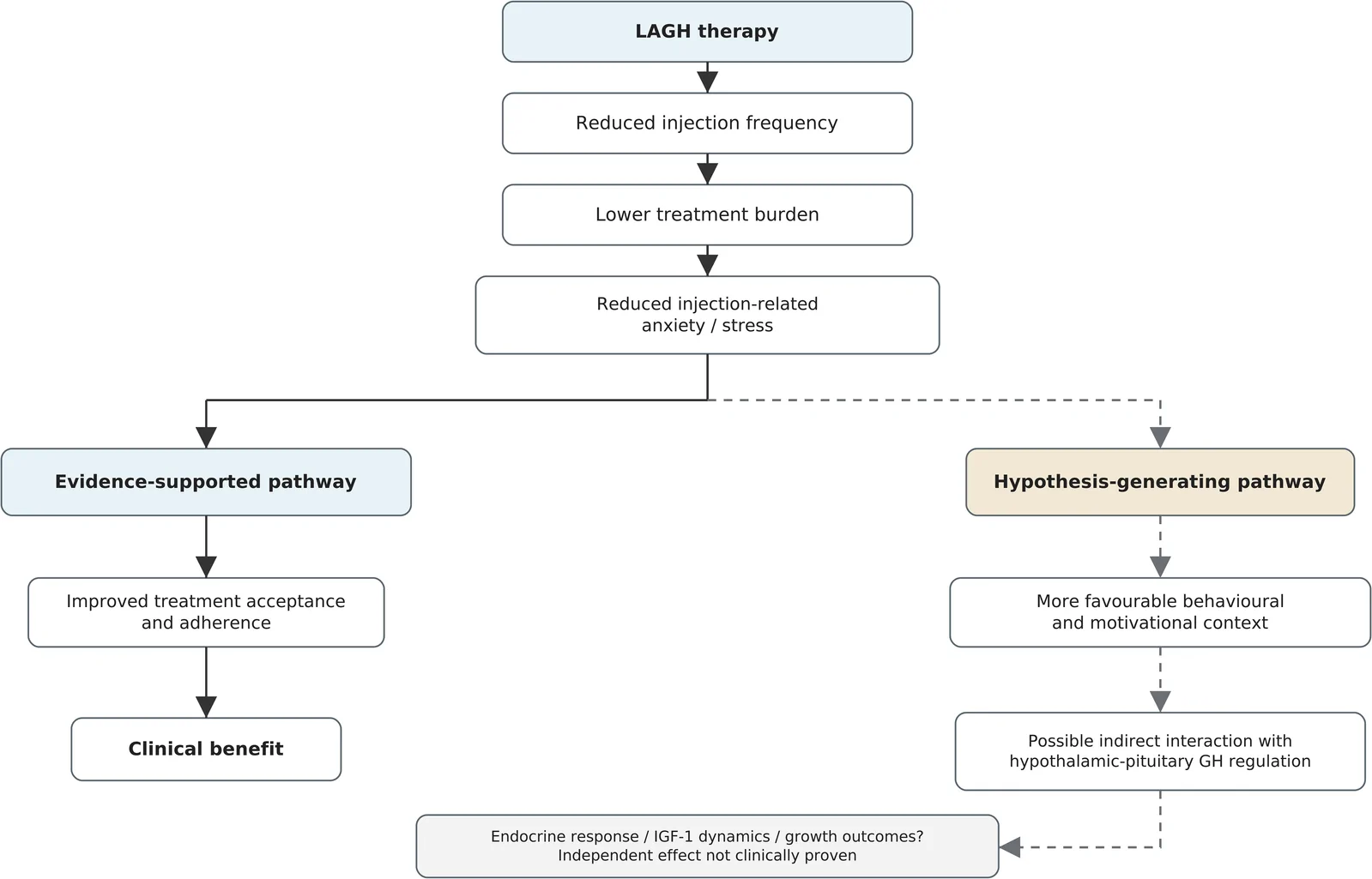
Evidence-supported and hypothesis-generating pathways linking long-acting growth hormone therapy, treatment experience, and growth hormone-related outcomes in paediatric growth hormone deficiency. indicate the best-supported clinical pathway, whereby reduced injection frequency and treatment burden may improve treatment acceptance, adherence, and clinical benefit. indicate the hypothesis-generating pathway whereby reduced stress and a more favourable behavioural and motivational context could indirectly interact with hypothalamic–pituitary growth hormone regulation. Direct clinical evidence that motivational state independently modifies endocrine response, insulin-like growth factor 1 dynamics, or growth outcomes during long-acting growth hormone therapy is currently lacking.

From a clinical perspective, reduced injection frequency, lower treatment burden, and improved adherence are established, evidence-supported advantages of LAGH therapy. Beyond adherence, reduced injection-related stress may theoretically improve the behavioural context of treatment. However, any downstream effects on hypothalamic–pituitary regulation or growth response remain speculative and have not been demonstrated in paediatric patients receiving LAGH. Available pharmacokinetic and clinical data support sustained IGF-1 exposure and favourable patient-reported outcomes during LAGH therapy, but they do not demonstrate that motivation-related neuroendocrine modulation independently alters endocrine response or growth outcomes beyond the better-established benefit of improved adherence (3, 4). Importantly, causality between motivational state and GH therapeutic response remains unproven, and improved adherence remains the most evidence-based mechanism underlying the clinical benefit of LAGH. Therefore, any reference to improved clinical outcomes in this review should be understood in relation to reduced injection burden and improved adherence, not as evidence of a proven neuroendocrine mechanism linking motivational state to superior growth response. The conceptual framework distinguishing evidence-supported clinical pathways from hypothesis-generating neuroendocrine pathways is summarised in **Figure 2**.

### Limitations of the current evidence base

Most of the evidence discussed regarding motivation-related neuroendocrine modulation is indirect, preclinical, or theoretical. Direct clinical evidence is currently lacking to demonstrate that such modulation independently alters therapeutic response to LAGH beyond the better-established benefit of improved adherence.

Much of the mechanistic evidence discussed in this review derives from animal models, *in vitro* experiments, or adult human physiological studies examining dopaminergic, ghrelinergic, opioid, stress-related, or hypothalamic modulation of GH secretion (13) (14, 15, 26, 28). Furthermore, paediatric LAGH evidence primarily supports reduced injection frequency, decreased treatment burden, adherence-related benefits, patient-reported treatment experience, pharmacokinetic/pharmacodynamic profiles, and clinical efficacy. It does not demonstrate an independent effect of motivation-related neuroendocrine modulation on therapeutic response. At present, improved adherence remains the most evidence-based mechanism linking LAGH therapy to clinical benefit.

Finally, this review does not provide a quantitative synthesis or formal grading of evidence. Its purpose is to generate a testable translational hypothesis and identify research directions that may clarify whether motivational and reward-related neuroendocrine pathways are associated with endocrine responses, growth outcomes, patient-reported treatment experiences, or adherence patterns during LAGH therapy.

### Future directions

To investigate whether motivational state is associated with endocrine response, growth-related endpoints, treatment experience, or adherence patterns during LAGH therapy, multidisciplinary translational studies are needed. Preclinical studies should integrate behavioural paradigms assessing motivational state, reward responsiveness, and stress exposure with endocrine and neurochemical assessments (7, 8, 26). These models may help test whether contextual behavioural variables are associated with GH pulsatility or IGF-1 generation; however, their relevance to responsiveness to long-acting GH formulations remains to be established.

Clinical research should incorporate validated measures of treatment-related stress, treatment experience, quality of life, and adherence alongside traditional endocrine and auxological endpoints (2–4). Adherence monitoring and patient-reported outcome measures may provide useful insights into the relationships among subjective treatment experience, adherence patterns, and biological responses. Longitudinal studies assessing growth velocity, body composition, and IGF-1 dynamics in relation to motivational profiles could help determine whether motivational state is associated with endocrine or growth-related endpoints, treatment experience, or adherence patterns during LAGH therapy.

## Conclusion

In conclusion, motivation-related neuroendocrine pathways, particularly those involving dopaminergic, opioid, stress-related, and metabolic signals, may provide a biologically plausible context for GH regulation, but their clinical relevance in paediatric LAGH therapy remains unproven. The reduced injection burden of LAGH formulations may improve treatment acceptance, reduce stress, and enhance adherence, which remains the most evidence-based mechanism underlying the clinical benefit of LAGH.

The broader hypothesis that motivational and reward-related states may have measurable relevance to endocrine responses, growth outcomes, or patient experience during LAGH therapy, beyond improved adherence, remains biologically plausible but is currently unproven. This perspective highlights the potential value of integrating behavioural neuroscience with endocrinology, while underscoring the need for direct translational studies before stronger clinical inferences are drawn. Future research should clarify whether motivational state is associated with endocrine response, growth outcomes, or patient experience in children with GHD treated with LAGH.
